# Supplementary material for: COVID-19 and digital transformation: developing an open experimental testbed for sustainable and innovative environments using Fuzzy Cognitive Maps
Source: F1000Res. 2021 Apr 1;10:264. [Version 1] doi: 10.12688/f1000research.51357.1 (PMC8314135; doi:10.12688/f1000research.51357.1)
Supplement: Peer review history

# Peer review: Reviewer response for version 1

**DOI:** 10.5256/f1000research.54517.r88763

**Published:** 2021-07-26

**Authors:** Chrysostomos D. Stylios

**Affiliations:** 1 Department of Informatics and Telecommunications, University of Ioannina, Ioannina, Greece

**Content**

 This paper proposes the application of Fuzzy Cognitive Maps (FCMs) to operably maps and simulate digital transformation in architecture and urban planning. By using this soft computing approach, the authors concluded that COVID-19 accelerates digitization to some extent.

**General Comments**

- The introduction must give a map of the paper. By the end of the introduction, reader should have a pretty good idea of what they're in for, and where they're going to end up. They should know what sections they're probably going to skip, and which ones they look forward to disagreeing with. So it would be advisable to write a brief organization of the paper.
- Second person pronouns are a good technique for giving instructions but it is not appropriate in academic or scientific writing.
- In page 3, the sentence “But there also other interesting solutions” could have been merged into one with the previous.
- Avoid repetition at the paper. Don't restate points you've already made.
- Use the first person singular pronoun appropriately, for example, to describe research steps or to state what you will do in a chapter or section. Do not use first person "I" to state your opinions or feelings.
- Table 2, 3,4, 5 and 6 could be combined in one table with 5 columns (one for each author).
- How the weight matrix was defined? In this paper the determination of the concepts is described extensively. More technical details about the weight matrix of each scenario should be mentioned (The causal interrelationships among concepts were declared using linguistic variables by an expert panel and a defuzzification method?).
- The FCM that have been developed (determination of the weight matrix) by the experts involve their subjectivity. Why was the application of a training algorithm for the weight adjustment not preferred?

**Overall Recommendations**

 There are many research papers that describe FCM development the more detailed explanation and various papers that investigate FCMs application and FCM in similar contexts so it is good to see work in this area and include them as references.

Is the rationale for developing the new method (or application) clearly explained?

Partly

Is the description of the method technically sound?

Partly

Are the conclusions about the method and its performance adequately supported by the findings presented in the article?

Partly

If any results are presented, are all the source data underlying the results available to ensure full reproducibility?

Yes

Are sufficient details provided to allow replication of the method development and its use by others?

Yes

Reviewer Expertise:

Soft computing, Fuzzy Cognitive Maps

I confirm that I have read this submission and believe that I have an appropriate level of expertise to confirm that it is of an acceptable scientific standard, however I have significant reservations, as outlined above.

# Peer review: Reviewer response for version 1

**DOI:** 10.5256/f1000research.54517.r87003

**Published:** 2021-06-21

**Authors:** Tamás Molnár

**Affiliations:** 1 Institute of Architecture, Faculty of Engineering and Information Technology, University of Pécs, Pécs, Hungary

The paper has generally a right structure. The description of the presented methodology is scientifically well based and clearly presented.

 Some small corrections would be needed to improve the paper:

- It is not the best solution to repeat a certain part of the abstract in the same way in the Introduction. Reformulating this part of the paper can make the introduction a little bit shorter.
- The abbreviation of BIM appears already in the earlier part of the Degree of automation chapter. The full name should be written there instead of the current place.
- It should be clarified what is the Münchener Kreis. At least an English translation should be provided, it would be even better to have a clarifying sentence.
- Instead of personal pronouns it is better to use the Author or passive voice e.g. the Author thinks or it is thought. In the Technologies chapter it is written “To my opinion”, in the Thought styles chapter it is written I fondly remember. These sentences should be corrected.
- The order of the tables are mixing up at the end of the paper. Table 16 and 17 should appear next to the text where it is mentioned, practically at the description of the scenarios. It is contradictory that Table 12 comes later than Table 16.
- There are a few spelling mistakes that should be corrected.

The overall evaluation of the paper is good and it is surely useful for further research in the challenging times of the current COVID-19 world.

Is the rationale for developing the new method (or application) clearly explained?

Yes

Is the description of the method technically sound?

Yes

Are the conclusions about the method and its performance adequately supported by the findings presented in the article?

Yes

If any results are presented, are all the source data underlying the results available to ensure full reproducibility?

Yes

Are sufficient details provided to allow replication of the method development and its use by others?

Yes

Reviewer Expertise:

Historical and contemporary development of architecture

I confirm that I have read this submission and believe that I have an appropriate level of expertise to confirm that it is of an acceptable scientific standard.

# Peer review: Reviewer response for version 1

**DOI:** 10.5256/f1000research.54517.r86940

**Published:** 2021-06-14

**Authors:** Peter Zeile

**Affiliations:** 1 Institute of Urban and Landscape Design, Karlsruhe Institute of Technology, Karlsruhe, Germany

F1000Research: COVID-19 and digital transformation: developing an open experimental testbed for sustainable and innovative environments using Fuzzy Cognitive Maps

**Content**

 This paper sketches a new approach using Fuzzy Cognitive Maps (FCMs) to operably maps and simulate digital transformation in architecture and urban planning. Today these processes are poorly understood. The paper introduces an experimental testbed in which economics, sustainability and social impacts were merged and develops three different digital transformation scenarios using FCMs

**General Comments**

 Is the ETSIE model *your own *developed model? If yes, please clarify it in the introduction and the abstract.

 For a better understanding, I would shorten the introduction or insert the section on FCMs as a separate chapter and structure it a little better with paragraphs. As we "jump" from the social component very quickly into the matter of FCMs, go into detail here with the advantages and disadvantages already, which may be easier to understand when viewed separately.

 Perhaps it would also be helpful to mention before the literature evaluation that the evaluation serves to "feed" the software so that the inserted tables are easier to understand at this point.

 Overall, the contribution "on the road" loses focus, since the actual question is whether we can better assess the consequences of digitalisation in urban planning and architecture. The topic is only taken up again in the discussion.

 Furthermore, as a reader who is not completely familiar with the matrix, one feels somewhat lost along the way; the individual steps are usually explained logically, but the big picture is sometimes not visible. Perhaps an exemplary workflow or flow chart would be useful, what data comes in where, which transformations and extracts are integrated into the Mental Modeler software, and what does that mean in the results if I am +0.50 "better".

 What the contribution does, however, is to summarise the framework conditions in the scenarios in such a way that the respective interactions can be (better?) assessed. These results are a good basis for discussing guiding principles, visions and goals at the political level.

 Of course, the question is always how much "trust" decision-makers have in such software that the consequences will occur when I turn screw X, (e.g. that the degree of digitalisation will decrease). and I think this is exactly where the article could sharpen the process or the "how", as described above with a flow chart.

 It would also be very helpful to have a few more specific statements on architecture and urban planning.

**In Details**

 Introduction: Please add that they are in the context of the German digital transformation, the ministers are probably not familiar to the international audience.

 "A study by BRZ Deutschland GmbH. names the following six IT trends in the construction industry." -> Which ones? See table 7?

**Overall Recommendations**

 Overall, an article with a lot of potentials, but with a little more structure and mapping based on the points mentioned above are still needed.

Is the rationale for developing the new method (or application) clearly explained?

Partly

Is the description of the method technically sound?

Yes

Are the conclusions about the method and its performance adequately supported by the findings presented in the article?

Partly

If any results are presented, are all the source data underlying the results available to ensure full reproducibility?

Yes

Are sufficient details provided to allow replication of the method development and its use by others?

Yes

Reviewer Expertise:

Urban Planning, Digital Methods in Urban Planning and Architecture

I confirm that I have read this submission and believe that I have an appropriate level of expertise to confirm that it is of an acceptable scientific standard, however I have significant reservations, as outlined above.
